# Angiogenic T Cells: Potential Biomarkers for the Early Diagnosis of Interstitial Lung Disease in Autoimmune Diseases?

**DOI:** 10.3390/biomedicines10040851

**Published:** 2022-04-05

**Authors:** Verónica Pulito-Cueto, Sara Remuzgo-Martínez, Fernanda Genre, Belén Atienza-Mateo, Víctor M. Mora-Cuesta, David Iturbe-Fernández, Leticia Lera-Gómez, Javier Rodriguez-Carrio, Diana Prieto-Peña, Virginia Portilla, Ricardo Blanco, Alfonso Corrales, Oreste Gualillo, José M. Cifrián, Raquel López-Mejías, Miguel A. González-Gay

**Affiliations:** 1 Research Group on Genetic Epidemiology and Atherosclerosis in Systemic Diseases and in Metabolic Bone Diseases of the Musculoskeletal System, IDIVAL, 39011 Santander, Spain; veronica_pulito_cueto@hotmail.com (V.P.-C.); sara.r.mtz@gmail.com (S.R.-M.); fernandagenre@gmail.com (F.G.); mateoatienzabelen@gmail.com (B.A.-M.); victormanuel.mora@scsalud.es (V.M.M.-C.); diturfer@gmail.com (D.I.-F.); letizialera@hotmail.com (L.L.-G.); diana.prieto.pena@gmail.com (D.P.-P.); virgiportilla@hotmail.com (V.P.); ricardo.blanco@scsalud.es (R.B.); afcorralesm@hotmail.com (A.C.); josemanuel.cifrian@scsalud.es (J.M.C.); 2 Department of Rheumatology, Hospital Universitario Marqués de Valdecilla, 39008 Santander, Spain; 3 Department of Pneumology, Hospital Universitario Marqués de Valdecilla, 39008 Santander, Spain; 4 Department of Functional Biology, Immunology Area, Faculty of Medicine, Universidad de Oviedo, 33006 Oviedo, Spain; javiercarrio@hotmail.com; 5 SERGAS (Servizo Galego de Saude) and IDIS (Instituto de Investigación Sanitaria de Santiago), NEIRID Lab. (Neuroendocrine Interactions in Rheumatology and Inflammatory Diseases), Research Laboratory 9, Santiago University Clinical Hospital, 15706 Santiago de Compostela, Spain; orestegualillo@gmail.com; 6 School of Medicine, Universidad de Cantabria, 39011 Santander, Spain; 7 Department of Medicine and Psychiatry, Universidad de Cantabria, 39011 Santander, Spain; 8 Cardiovascular Pathophysiology and Genomics Research Unit, School of Physiology, Faculty of Health Sciences, University of the Witwatersrand, Johannesburg 2050, South Africa

**Keywords:** angiogenic T cells, autoimmune disease, interstitial lung disease, systemic sclerosis, rheumatoid arthritis, biomarkers

## Abstract

(1) Background: We explored, for the first time, the contribution of angiogenic T cells (TAng) in interstitial lung disease associated to autoimmune disease (AD-ILD^+^) as potential biomarkers of the disease, evaluating their role in the underlying vasculopathy and lung fibrosis. Additionally, the relationship of TAng with clinical manifestations and cellular and molecular endothelial dysfunction-related biomarkers was assessed. (2) Methods: We included 57 AD-ILD^+^ patients (21 with rheumatoid arthritis (RA)-ILD^+^, 21 with systemic sclerosis (SSc)-ILD^+^ and 15 with other AD-ILD^+^) and three comparative groups: 45 AD-ILD^−^ patients (25 RA-ILD^−^ and 20 SSc-ILD^−^); 21 idiopathic pulmonary fibrosis (IPF) patients; 21 healthy controls (HC). TAng were considered as CD3^+^CD184^+^CD31^+^ by flow cytometry. (3) Results: A similar TAng frequency was found between AD-ILD^+^ and IPF, being in both cases lower than that observed in AD-ILD^−^ and HC. A lower TAng frequency was associated with negative Scl-70 status and lower FEV1/FVC ratio in SSc-ILD^+^, as well as with men in RA-ILD^+^ and non-specific interstitial pneumonia radiological pattern in other AD-ILD^+^. No relationship between TAng and endothelial progenitor cells, endothelial cells and vascular endothelial growth factor gene expression and protein levels was disclosed. (4) Conclusions: Our findings suggest TAng as potential biomarkers for the early diagnosis of ILD in AD.

## 1. Introduction

Interstitial lung disease (ILD) is a common and potentially life-threatening complication in patients with autoimmune diseases (AD), mainly in those with systemic sclerosis (SSc) and rheumatoid arthritis (RA) [1,2,3,4,5]. Early diagnosis of AD-ILD^+^ is sometimes challenging due to the potential absence of symptoms in early or mild disease and the similarity of radiological features with other entities involving the lung [1,2,3,4,5,6]. Currently, there is no established protocol to evaluate these patients, although several studies highlight the need for careful follow-up of these patients with both pulmonary function tests (PFTs) and high-resolution computed tomography (HRCT) [1,2,3,4,5,6,7]. In this sense, the early detection of pulmonary involvement is crucial to start an appropriate therapy and to avoid an irreversible damage to the lung in these patients [1,2,3,4,5,7,8]. A large body of evidence suggests that an impairment of vascular endothelium is a characteristic hallmark of the initial phase in these inflammatory diseases, ultimately resulting in a constitutive activation of fibroblasts in various organs, predominantly in the lung, leading to pulmonary fibrosis. In fact, the damage of the pulmonary endothelium has been described as one of the early key stages for the development of pulmonary lesions and the subsequent onset and progression of ILD in AD. However, the mechanisms underlying endothelial cell damage and defective repair remain incompletely understood in AD-ILD^+^ [1,3,9,10,11,12,13]. Endothelial progenitor cells (EPC) and endothelial cells (EC) are key cellular effectors in the homeostasis of the physiologic vascular network, and they have been described as an essential element of the endogenous vascular repair machinery in AD [14,15,16]. In this regard, we recently proposed EPC as biomarkers to identify the presence of ILD in patients with RA and SSc [14,15]. Moreover, it has been reported that a circulating cell population showing both M1 and M2 monocyte/macrophage surface markers characterizes SSc patients with lung involvement [17].

It has been described that a specific T cell population termed angiogenic T cells (TAng) cooperate with EPC in the endothelial repair function [18]. Since then, several studies have supported the notion that TAng promote the formation of new blood vessels and enhance the repair of damaged endothelium [9,18,19,20,21,22,23,24,25,26,27]. Furthermore, TAng exhibit a vasculogenic phenotype characterized by enhanced endothelial proliferation and may function by cell contact-dependent and paracrine mechanisms [9,18,28,29]. Specifically, TAng secrete a wide array of proangiogenic factors that have been implicated in AD-related angiogenic disturbances such as vascular endothelial growth factor (VEGF) [9,18,28,29]. Moreover, it has been demonstrated that TAng have migratory capacity towards the angiogenic chemoattractant VEGF secreted by injured endothelium [28,30]. Interestingly, altered TAng frequencies have been linked to RA [20,23,24], SSc [9,19], or to other AD [22,23,25,26,27]. Nevertheless, information on their role in the development of ILD in AD patients is scarce.

It has become apparent that the scarcity of useful markers for the early diagnosis of AD-ILD^+^ remains a problem that needs to be solved [1,2,4,8]. With respect to this, TAng may have an important role as biomarkers of endothelial damage in AD-ILD^+^. Accordingly, the main objective of this study was to determine, for the first time, the contribution of TAng in the pathogenesis of AD-ILD^+^ as potential biomarkers of the disease. For this purpose, we evaluated the role of TAng in the underlying vasculopathy of patients with AD-ILD^+^ and in the presence of lung fibrosis in these patients. Additionally, we also aimed to assess the relationship of TAng with AD-ILD^+^ clinical manifestations and endothelial dysfunction-related biomarkers at the cellular (EPC, CE) and molecular (*VEGF* mRNA expression and VEGF protein) level.

## 2. Materials and Methods

### 2.1. Study Population

Peripheral venous blood was collected from a total of 144 individuals. Specifically, 57 patients with AD-ILD^+^ were recruited: 21 with RA-ILD^+^, 21 with SSc-ILD^+^ and 15 with other AD-ILD^+^. Moreover, to assess the role of TAng in AD-ILD^+^, we recruited different comparative groups. A group of AD-ILD^−^ patients (*n* = 45) composed of 25 RA-ILD^−^ and 20 SSc-ILD^−^, another group of idiopathic pulmonary fibrosis (IPF) patients (*n* = 21), and 21 healthy controls (HC). Both patients and HC were recruited from the departments of Pneumology and Rheumatology of Hospital Universitario Marqués de Valdecilla (Santander, Spain).

Patients with AD had an underlying vasculopathy (clinically evident or not) and met the criteria established by the ACR/EULAR for the classification and diagnosis of each AD [31,32]. Pulmonary involvement was assessed in all the patients by HRCT images of the chest and PFTs. AD-ILD^−^ patients lacked lung involvement, whereas those with AD-ILD^+^ fulfilled the ATS/ERS criteria for ILD [33]. IPF patients fulfilled the ATS/ERS criteria [33]. HRCT patterns of ILD patients were stratified according to the criteria for usual interstitial pneumonia (UIP) pattern of the Fleischner Society [34]. Additionally, in SSc and IPF patients, pulmonary hypertension (PH) was diagnosed by transthoracic echocardiogram.

Demographic and clinical features of patients including sex, age, smoking history, duration of disease, PFTs, pulmonary involvement on HRCT and HRCT pattern, among others, were collected. The main characteristics of all the patients of the study group (RA-ILD^+^, SSc-ILD^+^ and other AD-ILD^+^) and the comparative groups (RA-ILD^−^, SSc-ILD^−^, IPF patients) are shown in Table 1. Furthermore, PH and other clinical manifestations of SSc patients were described in Table S1. HC did not present any history of autoimmune or lung diseases. Additionally, their mean age ± standard deviation (SD) was 41.2 ± 12.5 years, 33.3% of them were women, and 31.3 % were smokers.

All patients and HC gave their written informed consent to be included in the study. The procedures followed were in accordance with the ethical standards of the approved guidelines and regulations, according to the Declaration of Helsinki. The Ethics Committee of clinical research of Cantabria, Spain (2016.092) approved all experimental protocols.

### 2.2. Cell Quantification by Flow Cytometry

TAng quantification was analyzed by direct flow cytometry following a method previously described [24]. Briefly, cells obtained from 200 µL of peripheral blood were labelled with VioBlue-conjugated anti-CD3 (Miltenyi Biotec, Madrid, Spain), APC-conjugated anti-CD184 (Miltenyi Biotec, Madrid, Spain) and PE-conjugated anti-CD31 (Miltenyi Biotec, Madrid, Spain) monoclonal antibodies. In a further step, incubation with FACS lysing solution (BD Bioscience, San Jose, CA, USA) was performed to lyse red blood cells. After obtaining the white cell pellets, two washes with PBS were carried out. Finally, a CytoFLEX flow cytometer (Beckman Coulter, Brea, CA, USA) and the Cytexpert 2.3 analyzer (Beckman Coulter, Brea, CA, USA) were used to assess the labeled cells, acquiring approximately 3 × 10^4^ events per sample. CD3^+^ cells were gated and then assayed for the expression of CD184 and CD31 in the lymphocyte gate. TAng were considered as triple-positive for CD3, CD184 and CD31 (Figure S1) and expressed as percentage of cells in the lymphocyte gate.

EPC and EC frequencies were measured by flow cytometry following the method previously described [14,15]. EPC were considered as CD34^+^, CD45^low^, CD133^+^ and CD309^+^ cells and EC were defined as triple-negative for CD34, CD45 and CD133 and positive for CD309, following the nomenclature previously defined [14,15].

### 2.3. VEGF mRNA Expression

Total RNA was isolated from peripheral blood by a commercial RNA extraction kit (NucleoSpin RNA Blood Kit, Macherey-Nagel, Neumann-Neander-Str., Düren, Germany). The complementary DNA (cDNA) was obtained using iScript^TM^ Advanced cDNA Synthesis Kit for reverse transcription-quantitative real-time polymerase chain reaction (qPCR) (Bio-Rad, Madrid, Spain). qPCR was performed in the thermocycler QuantStudio™ 7 Flex Real-Time PCR System (Applied Biosystems, Foster City, CA, USA) using SsoAdvanced^TM^ Universal SYBR^®^ Green Supermix (Bio-Rad, Madrid, Spain). All samples were assayed in duplicate and experimental control assays were included. The relative *VEGF* mRNA expression was analyzed by the comparative Ct method using GAPDH as housekeeping gene.

### 2.4. VEGF Serum Levels Determination

VEGF levels were measured in serum samples by a commercial quantitative colorimetric sandwich enzyme-linked immunosorbent assay (Reddot Biotech Inc., Kelowna, BC, Canada) as previously described [35].

### 2.5. Statistical Analyses

Data were reported as the number of individuals (n) and percentage (%) or mean ± SD depending on the type of data. Differences in TAng frequencies between two study groups were calculated and compared by Student’s *t*-test. To evaluate the implication of TAng in the underlying vasculopathy, we compared all patients with HC, while their role in fibrosis was analyzed by comparing patients with AD-ILD^+^, patients with AD-ILD^−^ and patients with IPF. Estimation of the Pearson’s correlation coefficient (r) was used to assess the relationship of TAng frequency with continuous variables. To evaluate the association of TAng frequency with categorical variables, we employed one-way ANOVA. Statistical significance was defined as *p*-values < 0.05. STATA statistical software 12/SE (Stata Corp., College Station, TX, USA) was used to perform all statistical analysis.

## 3. Results

### 3.1. TAng Play a Role in the Pathogenesis of AD-ILD^+^

**Figure 1:**
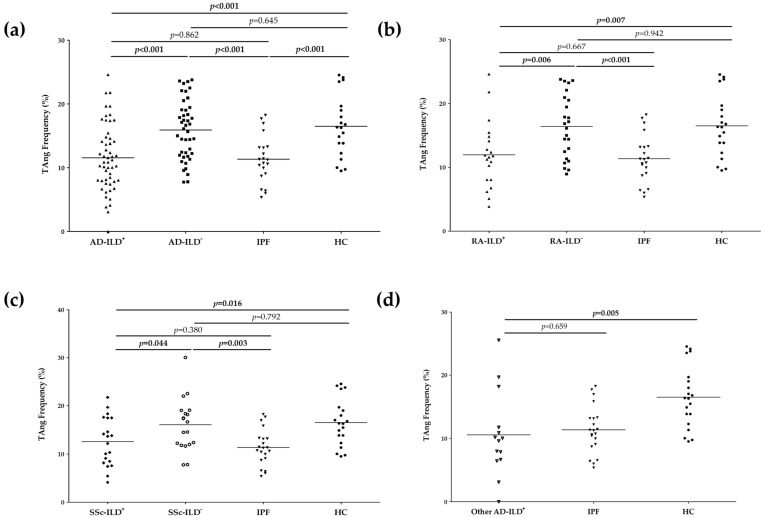
Differences in the frequency of TAng between all the study groups. Differences between patients with AD-ILD^+^, AD-ILD^−^, IPF and HC (**a**); patients with RA-ILD^+^, RA-ILD^−^, IPF y HC (**b**); patients with SSc-ILD^+^, SSc-ILD^−^, IPF and HC (**c**); and patients with other AD-ILD^+^, IPF and HC (**d**). TAng: angiogenic T cells; AD: autoimmune disease; RA: rheumatoid arthritis; ILD: interstitial lung disease; SSc: systemic sclerosis; IPF: idiopathic pulmonary fibrosis; HC: healthy controls. The horizontal bars indicate the mean value of each study group. Significant results are highlighted in bold.

First, we studied the role of TAng in the vasculopathy in AD-ILD^+^. Patients with AD-ILD^+^ showed a significantly lower frequency of TAng than HC (11.560 ± 5.242 vs. 16.500 ± 4.830, *p* < 0.001, Figure 1a and Table S2). It was also the case when IPF patients were compared with HC (11.340 ± 3.732 vs. 16.500 ± 4.830, *p* < 0.001, Figure 1a and Table S2). However, similar frequencies of TAng in patients with AD-ILD^−^ and HC were found (Figure 1a and Table S2). The same findings were seen when patients were stratified according to the underlying AD. In particular, frequencies of TAng were significantly decreased in patients with RA-ILD^+^ and SSc-ILD^+^ in relation to HC (11.950 ± 5.234 vs. 16.500 ± 4.830, *p* = 0.007 and 12.570 ± 5.052 vs. 16.500 ± 4.830, *p* = 0.016, respectively), unlike patients with RA-ILD^−^ and SSc-ILD^−^ who showed no differences with HC (Figure 1b,c and Table S2). Furthermore, patients with other AD-ILD^+^ displayed a lower frequency of TAng than HC (10.560 ± 6.684 vs. 16.500 ± 4.830, *p* = 0.005, Figure 1d and Table S2).

In a second step, we evaluated the implication of TAng in the presence of fibrosis in AD-ILD^+^. TAng frequencies were similar between patients with AD-ILD^+^ and those with IPF, while these frequencies were significantly lower in relation to those with AD-ILD^−^ (11.560 ± 5.242 vs. 15.920 ± 4.612, *p* < 0.001 and 11.340 ± 3.732 vs. 15.920 ± 4.612, *p* < 0.001, respectively, Figure 1a and Table S2). Specifically, patients with RA-ILD^+^ exhibited significantly lower TAng frequencies than those with RA-ILD^−^ (11.950 ± 5.234 vs. 16.400 ± 4.926, *p* = 0.006), but no differences were observed when they were compared to patients with IPF (Figure 1b and Table S2). Moreover, a significant increase in the frequency of TAng was seen in patients with RA-ILD^−^ when compared to those with IPF (16.400 ± 4.926 vs. 11.340 ± 3.732, *p* < 0.001, Figure 1c and Table S2). Patients with SSc-ILD^+^ and IPF had the same frequencies, which were significantly lower than those observed in patients with SSc-ILD^−^ (12.570 ± 5.052 vs. 16.070 ± 5.420, *p* = 0.044 and 11.340 ± 3.732 vs. 16.070 ± 5.420, *p* = 0.003, respectively, Figure 1c and Table S2). Likewise, TAng frequencies of patients with other AD-ILD^+^ were similar to the frequency of those with IPF (Figure 1d and Table S2).

### 3.2. TAng Are Associated with Demographic and Clinical Features of RA-ILD^+^, SSc-ILD^+^ and Other AD-ILD^+^

Regarding RA-ILD^+^ patients, men had significantly lower TAng frequencies than women (9.75 ± 4.12 vs. 16.44 ± 5.97, *p* < 0.01, Table 2), though no relationship was disclosed between these cells and the duration of RA, C-reactive protein (CRP), erytrocyte sedimentation rate (ESR) or PFTs. No differences were found in the frequency of TAng when patients with RA-ILD^+^ were stratified according to smoking history, rheumatoid factor/anti-cyclic citrullinated peptide antibodies status or HRCT pattern (Table 2).

With respect to SSc-ILD^+^ patients, a positive correlation between the frequency of TAng and the forced expiratory volume in one second (FEV1)/forced vital capacity (FVC) ratio was observed in these patients (*r* = 0.48; *p* = 0.03, Table 3). Anti-Scl70 negative SSc-ILD^+^ patients presented lower TAng frequencies compared to anti-Scl70 positive patients (10.30 ± 5.09 vs. 15.73, *p* = 0.03, Table 3). No significant relationship was found between the frequency of TAng and SSc duration, CRP or ESR (Table 3). The same results were obtained when SSc-ILD^+^ patients were stratified according to sex, smoking history, anti-nuclear antibodies/anti-centromere antibodies status, presence of pulmonary hypertension or HRCT pattern (Table 3).

In relation to patients with other AD-ILD^+^, differences in the frequency of TAng were found when these patients were stratified according to the HRCT pattern (Table 4). Specifically, patients who presented a NSIP pattern had lower TAng frequencies than those with an UIP pattern (6.43 ± 3.99 vs. 15.11 ± 7.69, *p* = 0.03, Table 4). Nonetheless, no associations of PFTs with TAng were noted in these patients (Table 4). Similarly, we did not disclose an association with TAng frequency when these patients with other AD-ILD^+^ were analyzed according to sex or smoking history (Table 4).

### 3.3. No Relationship of TAng with Biomarkers of Endothelial Dysfunction in the Whole Cohort of AD-ILD^+^

TAng frequency did not show correlation with EPC or EC frequency in AD-ILD^+^ patients (Table S3). Likewise, no association between TAng frequency and VEGF, either at mRNA expression or at protein level, was observed (Table S3).

## 4. Discussion

Growing evidence indicates that vascular abnormalities constitute the early phase in the pathogenesis of AD-ILD^+^ [1,3,9,10,11,12]. To the best of our knowledge, this is the first study exploring the implication of TAng, a crucial player in endothelial repair [18], in the pathogenic processes of lung fibrosis and vasculopathy in patients with AD-ILD^+^.

The present findings provide the first evidence that TAng may be a relevant factor involved in the processes of lung fibrosis. This idea is supported by the decrease in TAng in patients with AD-ILD^+^ compared to those with AD-ILD^−^. In line with this notion, patients with RA-ILD^+^ and SSc-ILD^+^ showed a decrease in TAng compared to RA-ILD^−^ and SSc-ILD^−^ patients, respectively, demonstrating the same behavior of TAng regardless of the underlying AD. In keeping with our results, a previous study showed different frequencies of TAng in systemic lupus erythematous (SLE) depending on the presence or absence of a renal involvement, one of the most severe comorbidities of SLE [27]. Interestingly, our work disclosed that patients with IPF presented TAng frequencies similar to those with AD-ILD^+^ and lower than AD-ILD^−^ patients. Accordingly, we disclosed that TAng were decreased in all the individuals with a lung involvement, including both AD-ILD^+^ and IPF patients, compared to those unaffected by this condition, highlighting the contribution of TAng in the pulmonary complications. Therefore, a reduction in TAng may indicate the presence of lung fibrosis. Based on our results and given that the development of ILD is one of the main causes of mortality in AD patients [1,2,4], TAng could be used as novel biomarkers for the early diagnosis of AD-ILD^+^.

Following the same line of evidence, both patients with AD-ILD^+^ and IPF showed a remarkable decrease in TAng frequency when compared to HC. In accordance with our results, it has been previously reported that TAng diminish in response to vascular disease in other disorders [23,24,25,29,30,36,37]. Furthermore, our data showed that TAng frequency in AD-ILD^−^ patients, in particular in RA-ILD^−^ and SSc-ILD^−^ patients, was not different from HC, as disclosed in other rheumatic diseases [9,23,26,27]. Consequently, we could speculate that the decrease in circulating TAng in AD-ILD^+^ and IPF patients occurs because they are migrating to the site of lung injury to repair the endothelium, constituting a marker of lung vasculopathy.

In the present study, we also disclosed a relationship of TAng with some characteristics of our patients with AD-ILD^+^. Notably, we found a lower frequency of TAng in men with RA-ILD^+^, which seems to be expected considering that the male sex is a known RA-ILD^+^ risk factor [1]. Paradoxically, a higher TAng frequency was observed in Scl-70-positive when compared with Scl-70-negative SSc-ILD^+^ patients. Additionally, a higher TAng frequency was associated with a higher FEV1/FVC ratio in SSc-ILD^+^ patients. Since the Scl-70 antibody is a risk factor for the development of ILD in patients with SSc and a decrease in FVC is used as a routine measure to assess disease progression in fibrotic ILD [5], it is possible that the relative TAng increases in these two situations in SSc-ILD^+^ patients may be due to a compensatory mechanism in response to vascular damage. It is worth mentioning that patients with other AD-ILD^+^ who presented NSIP pattern had the lowest TAng frequencies. This is in line with the fact that NSIP is the predominant pattern in AD-ILD^+^ [1,2,8,33,34,38].

Finally, a relationship of TAng with EPC or EC was not found in peripheral blood of our AD-ILD^+^ patients. These results are in keeping with other studies in which a lack of association was described in patients with RA and diabetes mellitus [24,37]. It is possible that the cooperation of TAng and EPC may take place when they are already in the damaged tissues and not at the blood level. Additionally, we did not find an association of TAng with VEGF. This finding may be explained by the fact that VEGF secretion is regulated by many different factors in AD-ILD^+^ or even at different molecular levels.

In conclusion, our findings suggest, for the first time, that TAng play a relevant role in the underlying lung vasculopathy and fibrosis, being potential biomarkers of ILD in patients with AD. Therefore, the assessment of TAng could help to establish an earlier diagnosis of AD-ILD^+^. This may favor the use of appropriate therapy in earlier stages of the disease, preventing progression to an irreversible pulmonary process and, ultimately, contributing to improving the survival of patients with AD.

The results of this work were partially presented at the American College of Rheumatology (ACR) 2021 Congress (abstract no. 1508) (View Abstract and Citation Information Online https://acrabstracts.org/abstract/decrease-of-angiogenic-t-cells-associated-to-the-presence-of-interstitial-lung-disease-in-patients-with-connective-tissue-diseases/ (accessed on 4 April 2022)), European Alliance of Associations for Rheumatology (EULAR) 2021 Congress (abstract no. AB0026) (View Abstract and Citation Information Online https://ard.bmj.com/content/80/Suppl_1/1046.3 (accessed on 4 April 2022)) and European Respiratory Society (ERS) Virtual Congress (abstract no. 27636) (view abstract and citation information online: https://erj.ersjournals.com/content/58/suppl_65/PA3620 (accessed on 4 April 2022)).

## Figures and Tables

**Table 1:** Main characteristics of all the patients of the study objective groups and the comparative groups.

	Study Objective Groups			Comparative Groups		
	RA-ILD^+^ *n* = 21	SSc-ILD^+^ *n* = 21	Other AD-ILD^+^ *n* = 15	RA-ILD^−^ *n* = 25	SSc-ILD^−^ *n* = 20	IPF *n* = 21
Sex (women), *n* (%)	9 (45.9)	13 (61.9)	5 (33.3)	15 (60.0)	18 (90.0)	7 (33.3)
Age at study, mean ± SD, years	66.5 ± 10.1	60.3 ± 7.0	62.0 ± 10.1	60.1 ± 11.8	56.6 ± 15.4	69.2 ± 10.0
Smoking ever, *n* (%)	13 (65.0)	11 (52.4)	11 (73.3)	13 (52.0)	11 (55.0)	16 (76.2)
**Pulmonary function tests**						
FVC (% predicted), mean ± SD	95.2 ± 24.1	88.4 ± 27.1	88.3 ± 28.8	99.2 ± 16.0	106.6 ± 15.9	84.9 ± 14.7
FEV1 (% predicted), mean ± SD	92.2 ± 21.0	87.3 ± 25.6	88.7 ± 27.6	94.9 ± 22.0	101.9 ± 17.8	87.3 ± 19.6
FEV1/FVC (% predicted), mean ± SD	77.8 ± 9.1	79.7 ± 5.5	79.7 ± 4.6	93.6 ± 12.3	79.2 ± 9.9	79.7 ± 7.8
DLCO (% predicted), mean ± SD	43.3 ± 15.9	47.5 ± 19.5	44.6 ± 14.6	79.9 ± 20.0	71.5 ± 15.3	43.6 ± 18.4
**HRCT**						
Pulmonary involvement on HRCT	21 (100.0)	21 (100.0)	15 (100.0)	0 (0.0)	0 (0.0)	21 (100.0)
UIP pattern, *n* (%)	11 (52.4)	3 (14.3)	4 (26.7)	-	-	21 (100.0)
Probable UIP pattern, *n* (%)	2 (9.5)	3 (14.3)	5 (33.3)	-	-	0 (0.0)
NSIP pattern, *n* (%)	7 (33.3)	14 (66.7)	6 (40.0)	-	-	0 (0.0)
Non-NSIP pattern, *n* (%)	1 (4.8)	1 (4.7)	0 (0.0)	-	-	0 (0.0)
**Received therapies**						
csDMARDs *n* (%)	17 (81.0)	16 (76.2)	2 (13.3)	13 (52)	12 (60.0)	0 (0.0)
bDMARDs, *n* (%)	15 (71.4)	7 (33.3)	3 (20.0)	2 (8)	2 (10.0)	0 (0.0)
Antifibrotic drugs, *n* (%)	0 (0.0)	0 (0.0)	0 (0.0)	0 (0.0)	0 (0.0)	9 (42.9)

RA: rheumatoid arthritis; ILD: interstitial lung disease; SSc: systemic sclerosis; AD: autoimmune disease; IPF: idiopathic pulmonary fibrosis; SD: standard deviation; FVC: forced vital capacity; FEV1: forced expiratory volume in one second; DLCO: diffusing capacity of the lung for carbon monoxide; HRCT: high resolution computed tomography; UIP: usual interstitial pneumonia; NSIP: non-specific interstitial pneumonia; csDMARDs: conventional synthetic disease-modifying anti-rheumatic drugs; bDMARDs: biologic disease-modifying anti-rheumatic drugs.

**Table 2:** Relationship of TAng frequency with characteristics of RA-ILD^+^ patients.

Variable	*r*	*p*
**Duration of RA (years)**	−0.16	0.50
**CRP (mg/dL)**	0.02	0.94
**ESR (mm/1st hour)**	−0.18	0.44
**FVC (% predicted)**	0.13	0.58
**FEV1 (% predicted)**	0.18	0.43
**FEV1/FVC (% predicted)**	0.18	0.44
**DLCO (% predicted)**	0.15	0.62
**Category**	**Mean ± SD**	***p***
**Men**	**9.75 ± 4.12**	**<0.01**
**Women**	**16.44 ± 5.97**	
**Non-Smoker**	15.50 ± 5.63	0.24
**Smoker**	12.02 ± 5.86	
**RF^−^**	13.59 ± 3.29	0.79
**RF^+^**	12.52 ± 6.50	
**UIP HRCT Pattern**	12.40 ± 6.34	0.76
**NSIP HRCT Pattern**	13.36 ± 6.55	

TAng: angiogenic T cells; RA: rheumatoid arthritis; ILD: interstitial lung disease; CRP: C-reactive protein; ESR: erythrocyte sedimentation rate; FVC: forced vital capacity; FEV1: forced expiratory volume in one second; DLCO: diffusing capacity of the lung for carbon monoxide; SD: standard deviation; RF: rheumatoid factor; UIP: usual interstitial pneumonia; HRCT: high resolution computed tomography; NSIP: non-specific interstitial pneumonia. Significant results are highlighted in bold.

**Table 3:** Relationship of TAng frequency with characteristics of SSc-ILD^+^ patients.

Variable	*r*	*p*
**Duration of SSc disease (years)**	0.04	0.86
**CRP (mg/dL)**	0.31	0.22
**ESR (mm/1st hour)**	−0.17	0.51
**FVC (% predicted)**	−0.06	0.79
**FEV1 (% predicted)**	−0.02	0.94
**FEV1/FVC (% predicted)**	**0.48**	**0.03**
**DLCO (% predicted)**	−0.06	0.77
**Category**	**Mean ± SD**	***p***
**Men**	10.30 ± 5.67	0.07
**Women**	15.02 ± 5.25	
**Non-Smoker**	14.97 ± 5.20	0.19
**Smoker**	11.64 ± 6.04	
**ATA (Scl70)^−^**	**10.30 ± 5.09**	**0.03**
**ATA (Scl70)^+^**	**15.73 ± 5.44**	
**Non-Pulmonary hypertension**	12.38 ± 6.08	0.37
**Pulmonary hypertension**	15.86 ± 5.15	
**NSIP HRCT Pattern**	13.52 ± 6.61	0.70
**UIP HRCT Pattern**	12.38 ± 4.28	

TAng: angiogenic T cells; SSc: systemic sclerosis; ILD: interstitial lung disease; CRP: C-reactive protein; ESR: erythrocyte sedimentation rate; FVC: forced vital capacity; FEV1: forced expiratory volume in one second; DLCO: diffusing capacity of the lung for carbon monoxide; SD: standard deviation; ATA: anti-topoisomerase I antibodies; NSIP: non-specific interstitial pneumonia; HRCT: high resolution computed tomography; UIP: usual interstitial pneumonia. Significant results are highlighted in bold.

**Table 4:** Relationship of TAng frequency with characteristics of other AD-ILD^+^ patients.

Variable	*r*	*p*
**FVC (% predicted)**	−0.27	0.32
**FEV1 (% predicted)**	−0.27	0.32
**FEV1/FVC (% predicted)**	0.15	0.59
**DLCO (% predicted)**	−0.36	0.27
**Category**	**Mean ± SD**	***p***
**Men**	11.92 ± 8.97	0.84
**Women**	11.05 ± 4.92	
**Non-Smoker**	17.83 ± 7.30	0.06
**Smoker**	9.38 ± 6.74	
**NSIP HRCT Pattern**	**6.43 ± 3.99**	**0.03**
**UIP HRCT Pattern**	**15.11 ± 7.69**	

TAng: angiogenic T cells; AD: autoimmune disease; ILD: interstitial lung disease; FVC: forced vital capacity; FEV1: forced expiratory volume in one second; DLCO: diffusing capacity of the lung for carbon monoxide; SD: standard deviation; NSIP: non-specific interstitial pneumonia; HRCT: high resolution computed tomography; UIP: usual interstitial pneumonia. Significant results are highlighted in bold.

## Data Availability

All data generated or analyzed during this study are included in this published article.

## Supplementary Materials

The following supporting information can be downloaded at: https://www.mdpi.com/article/10.3390/biomedicines10040851/s1, Figure S1: Representative dot-blots of the strategy used to quantify of TAng by flow cytometry; Table S1: Clinical manifestations of patients with SSc-ILD^+^ and SSc-ILD^−^; Table S2: Frequency of TAng (%) in all the individuals included in the study; Table S3: Detailed information of cellular and molecular endothelial dysfunction-related biomarkers in the whole cohort of patients with AD-ILD^+^.
