# Supplementary material for: Angiogenic T Cells: Potential Biomarkers for the Early Diagnosis of Interstitial Lung Disease in Autoimmune Diseases?
Source: Biomedicines. 2022 Apr 5;10(4):851. doi: 10.3390/biomedicines10040851 (PMC9026324; doi:10.3390/biomedicines10040851)
Supplement: Supplementary file 1 [file biomedicines-10-00851-s001.zip › biomedicines-1655057-supplementary.pdf]

biomedicines

Article

Angiogenic T cells: potential biomarkers for the early diagnosis of interstitial lung disease in autoimmune diseases?

Verónica Pulito-Cueto[1*], Sara Remuzgo-Martínez[1*], Fernanda Genre[1*], Belén Atienza-Mateo[1,2], Víctor M. Mora-Cuesta[1,3], David Iturbe-Fernández[1,3], Leticia Lera-Gómez[1], Javier Rodriguez-Carrio[4], Diana Prieto-Peña[1,2], Virginia Portilla[1,2], Ricardo Blanco[1,2], Alfonso Corrales[1,2], Oreste Gualillo[5], José M. Cifrián[1,3,6], Raquel López-Mejías[1] and Miguel A. González-Gay[7,8**]**

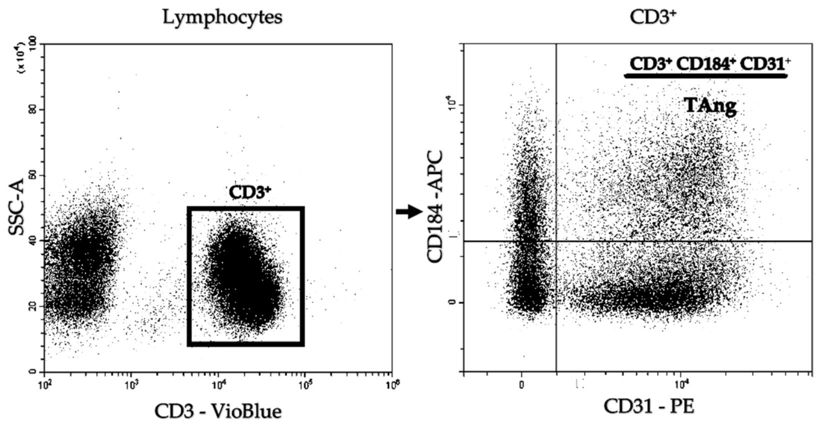

Supplementary Figure S1. Representative dot-blots of the strategy used to quantify of TAng by flow cytometry. $CD3^+$ were gated and then assayed for expression of CD184 and CD31 in the lymphocyte gate. TAng were considered as $CD3^+$/$CD184^+$/$CD31^+$ cells in the lymphocyte gate. Images obtained from Cytexpert 2.3 analyzer.

biomedicines

Supplementary Table S1. Clinical manifestations of patients with SSc-ILD$^+$ and SSc-ILD$^-$.

	**SSc-ILD$^+$ patients *n* = 21**	**SSc-ILD$^-$ patients *n* = 20**
Pulmonary hypertension, *n* (%)	3 (15.8)	0 (0.0)
Renal impairment, *n* (%)	1 (4.8)	1 (5.0)
Cardiac involvement, *n* (%)	6 (28.6)	1 (5.0)
Raynaud's phenomenon, *n* (%)	21 (100.0)	20 (100.0)
Esophageal dysfunction, *n* (%)	12 (57.1)	5 (25.0)
Calcinosis, *n* (%)	0 (0.0)	6 (30.0)
Synovitis, *n* (%)	6 (28.6)	6 (30.0)

SSc: systemic sclerosis; ILD: interstitial lung disease.

Supplementary Table S2. Frequency of TAng (%) in all the individuals included in the study.

		Mean ± SD
Study Objective Groups	**AD-ILD$^+$**	11.560 ± 5.242
	**RA-ILD$^+$**	11.950 ± 5.234
	**SSc-ILD$^+$**	12.570 ± 5.052
	**Other AD-ILD$^+$**	10.560 ± 6.684
Comparative Groups	**AD-ILD$^-$**	15.920 ± 4.612
	**RA-ILD$^-$**	16.400 ± 4.926
	**SSc-ILD$^-$**	16.070 ± 5.420
	**IPF**	11.340 ± 3.732
	**HC**	16.500 ± 4.830

TAng: angiogenic T cells; SD: standard deviation; AD: autoimmune disease; ILD: interstitial lung disease; RA: rheumatoid arthritis; SSc: systemic sclerosis; IPF: idiopathic pulmonary fibrosis; HC: healthy controls.

biomedicines

Supplementary Table S3. Detailed information of cellular and molecular endothelial dysfunction-related biomarkers in the whole cohort of patients with AD-ILD^{+}.

**EPC Frequency** (%) *(Mean ± SD)*	0.037 ± 0.017
**EC Frequency** (%) *(Mean ± SD)*	0.017 ± 0.019
***VEGF* mRNA expression** *(Mean ± SD)*	0.040 ± 0.069
**VEGF serum levels** *(pg/mL) (Mean ± SD)*	89.620 ± 63.840

AD: autoimmune disease; ILD: interstitial lung disease; EPC: endothelial progenitor cells; SD: standard deviation; EC: endothelial cells; VEGF: vascular endothelial growth factor.
